# Design of a New Catalyst, Manganese(III) Complex, for the Oxidative Degradation of Azo Dye Molecules in Water Using Hydrogen Peroxide

**DOI:** 10.3390/molecules29215217

**Published:** 2024-11-04

**Authors:** Raoudha Soury, Adel Elamri, Mabrouka El Oudi, Khalaf M. Alenezi, Mahjoub Jabli, Ahmed Al Otaibi, Abdulaziz A. Alanazi, Abuzar E. A. E. Albadri

**Affiliations:** 1 Department of Chemistry, College of Science, University of Hail, Ha’il 81451, Saudi Arabia; 2 Materials and Processes Research Unit, Tunisia National Engineering School of Monastir, University of Monastir, Monastir 5000, Tunisia; amri.adel2201@gmail.com; 3 Department of Chemistry, College of Science, Majmaah University, Al-Majmaah 11952, Saudi Arabia; m.jabli@mu.edu.sa; 4 Department of Chemistry, College of Science and Humanities in Al-Kharj, Prince Sattam bin Abdulaziz University, Al-Kharj 11942, Saudi Arabia; 5 Department of Physics, College of Science, Qassim University, Qassim 51452, Saudi Arabia

**Keywords:** chloro(*meso*-tetrakis(phenyl)porphyrin) manganese(III), *meso*-tetraphenylporphyrin, calmagite, degradation, H_2_O_2_

## Abstract

In the current work, chloro(*meso*-tetrakis(phenyl)porphyrin) manganese(III) [Mn(TPP)Cl] was synthesized following two steps: the preparation of *meso*-tetraphenylporphyrin (H_⁠2_TPP) and the insertion of manganese into the free porphyrin H_2_TPP. The compounds were characterized using SEM, FT-IR, UV, TGA/DTA, and XRD analyses. Manganese(III) *meso*-porphyrins exhibited hyper-type electronic spectra with a half-vacant metal orbital with symmetry, such as [dπ:dxz and dyz]. The thermal behavior of [Mn(TPP)(Cl)] changed (three-step degradation process) compared to the initial H_2_TPP (one-step degradation process), confirming the insertion of manganese into the core of the free porphyrin H_2_TPP. Furthermore, [Mn(TPP)Cl] was used to degrade calmagite (an azo dye) using H_2_O_2_ as an oxidant. The effects of dye concentration, reaction time, H_2_O_2_ dose, and temperature were investigated. The azo dye solution was completely degraded in the presence of [Mn(TPP)(Cl)]/H_2_O_2_ at pH = 6, temperature = 20 °C, C_0_ = 30 mg/L, and H_2_O_2_ = 40 mL/L. The computed low activation energy (Ea = 10.55 Kj/mol) demonstrated the efficiency of the proposed catalytic system for the azo dye degradation. Overall, based on the synthesis process and the excellent catalytic results, the prepared [Mn(TPP)Cl] could be used as an effective catalyst for the treatment of calmagite-contaminated effluents.

## 1. Introduction

The porphyrin skeleton is extensively utilized in a wide range of biological, physical, and catalytic systems, as evidenced by numerous reports [1,2,3,4,5,6]. Porphyrins and their derivatives are highly regarded due to their diverse applications [7,8]. This is attributed to the metal bound to the nitrogen atoms in the core of the porphyrin ring and the substituents attached to the tetrapyrrolic ring [9,10,11]. In fact, the inserted metals and the adjustment of the out-of-plane distance within the porphyrin cavity can regulate the properties of metallo-porphyrins. For these reasons, porphyrins and metallo-porphyrins have been extensively applied in drug delivery, biosensing, bio-imaging, industrial, photocatalytic, nonlinear optics, molecular photovoltaics, industrial, and analytical fields [12,13,14,15,16,17]. Moreover, it has been demonstrated that their photophysical and biological properties are altered when a transition metal like manganese (Mn) is inserted, changing the symmetry of the free porphyrins [18,19]. In particular, because of the coordination of the Mn and the range of oxidation states (+II, +III, or +IV), Mn–porphyrins exhibit higher activity than other porphyrinic complexes [20,21,22]. The Mn(III)–porphyrin complexes were the most investigated. Five- or six-coordinate bonds make up the Mn–porphyrin derivatives when the manganese is in the oxidation state +III. This has to do with the metal center’s surroundings and synchronization. Six-coordinated Mn(III)–porphyrin species are often low-spin, whereas five-coordinated derivatives of Mn(III)–porphyrin are typically high-spin [21,22]. The electronic characteristics of Mn(III)–porphyrin species with five coordinates differ entirely from those of other Mn(III)–porphyrin species, such as those from Fe(II), Co(II), Mn(II), and Ni(II). The UV–visible spectra of the high-spin Mn(III) derivatives with *meso*-porphyrins are of the d-hyper type.

The removal of pollutants from wastewater is crucial because of their harmful impact on ecosystems [23]. Specifically, synthetic dyes discharged into contaminated water bodies have been identified as carcinogenic and hazardous. Therefore, there is a significant focus on eliminating these dyes from aquatic systems [24]. The development of homogenous and heterogenous catalysts has garnered specific attention due to their diverse applications across various fields [25,26,27]. Among various methods reported in the literature, catalytic oxidation is a commonly employed method for treating contaminated water, as it has proven to be effective in removing organic dyes. This technique involves the use of hydrogen peroxide (H_2_O_2_) as an environmental oxidizing agent [26]. Research has shown that various catalysts can be used to decolorize dye solutions, making catalytic oxidation a versatile and efficient process for water treatment. For example, Abir et al. [27] investigated the degradation of Acid Orange 7 dye in aqueous solution using HFe_2.5_P_2_W_18_O_62_ 23·H_2_O as a catalyst and H_2_O_2_ as an oxidant. They found that, under optimum conditions, the efficiency of degradation could reach up to 100%. Following this, Samarghandi et al. [28] investigated the degradation of an azo dye (acid red 14) using a nano-catalytic system and in the presence of ultraviolet light. They reported a high removal efficiency (89.3–93.94%) of the catalytic system. Cao et al. [29] used Mn(II) ions to catalyze the oxidative degradation of calmagite, using H_2_O_2_ and 1,2-dihydroxybenzene-3,5-disulfonate as the co-catalyst. The percentage of degradation ranged from 91.1% to 96.0%. Karla et al. [30] studied the degradation of Allura Red AC solution by activating H_2_O_2_ with bicarbonate, using Co^2+^ ions as the catalyst. Under optimal conditions, the degradation reached 99.86%. In our previous work [31], we reported the degradation of calmagite using biopolymer-supported MnO_2_ and SnO_2_ in the presence of H_2_O_2_.

Porphyrins play a crucial role in catalysis, especially metallo-porphyrins, which have demonstrated enhanced catalytic activity in various chemical and photochemical processes. Studies have shown that porphyrinic compounds are effective in degrading organic dyes. For instance, a hybrid material consisting of magnetic CuFe_2_O_4_–porphyrin nanofibers was utilized as a photocatalyst to degrade rhodamine B dye [32]. Additionally, complex tetrakis(4-carboxyphenyl)–porphyrin nanofibers and ZnO nanoparticles were employed for the photocatalytic degradation of rhodamine B under sunlight irradiation [33]. Furthermore, a zinc–tetraphenylporphyrin complex was employed to photodegrade methylene blue using UV light [34]. Moreover, a Co(II) complex of tetrakis-5, 10, 15, 20 (4-hydroxyphenyl)porphyrin Co(II)TPHPP anchored to chloroacetylated poly (p-hydroxy styrene) was utilized for the catalytic decomposition of crystal violet using H_2_O_2_ [35].

Calmagite (2-hydroxy-1-(2-hydroxy-5-methylphenylazo)-4-naphthalenesulfonic acid) is a toxic and non-biodegradable azo dye compound. It has a half-life period of more than a year [36]. Motivated by this, we present herein a first report on the oxidative degradation of calmagite in water using H_2_O_2_ and chloro(*meso*-tetrakis(phenyl)porphyrin) manganese(III). Calmagite has azo groups as chromophore groups, which can be easily degraded using metallic complexes in combination with oxidizing agents. Analytical techniques including FT-IR, SEM, XRD, UV, and TGA/DTA were used to characterize the prepared compounds. Different experimental parameters were investigated, including temperature, time of reaction, starting dye concentration, and H_2_O_2_ dose. To better understand the oxidative degradation of calmagite, the experimental data were fitted to zero-, first-, and second-order kinetic equations. The thermodynamic parameters and the activation energy (Ea) were also calculated.

## 2. Results and Discussion

### 2.1. Material Characterization

#### 2.1.1. FT-IR Spectroscopy

**Figure 1:**
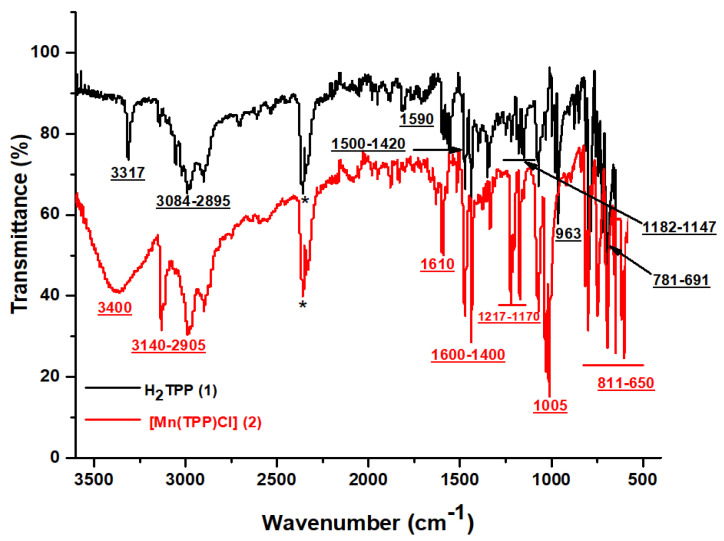
FT-IR spectra of H_2_TPP and [Mn(TPP)(Cl)]. (*: CO_2_ peak).

The FT–IR spectra of H_2_TPP and [Mn(TPP)Cl] are given in Figure 1. The IR spectrum exhibits the bands characteristic of *meso*-porphyrin H_2_TPP. Indeed, the vibration band ν (NH) for the free porphyrin is detected at around 3317 cm^−1^. The C–H groups of H_2_TTP moiety are seen in the range 2895–3084 cm^−1^. C=N and C=C stretching frequencies are observed in the range 1500–1420 cm^−1^ and 1590 cm^−1^, respectively. The absorption bands at 1147 and 1182 cm^−1^ are assigned to vibration ν (C-C). The strong band attributed to the bending vibration of the CCH moieties of the porphyrin core is centered around 963 cm^−1^. The absorption bands between 781 and 691 cm^−1^ are attributed to the ν (C-C) phenyl group [37,38]. For the [Mn(TPP)Cl] spectrum, the disappearance of ν (NH) and the slight displacement of the other bands prove the insertion of the Mn metal in the porphyrinic core [38,39].

#### 2.1.2. UV–Visible Absorption and Optical Gap

**Figure 2:**
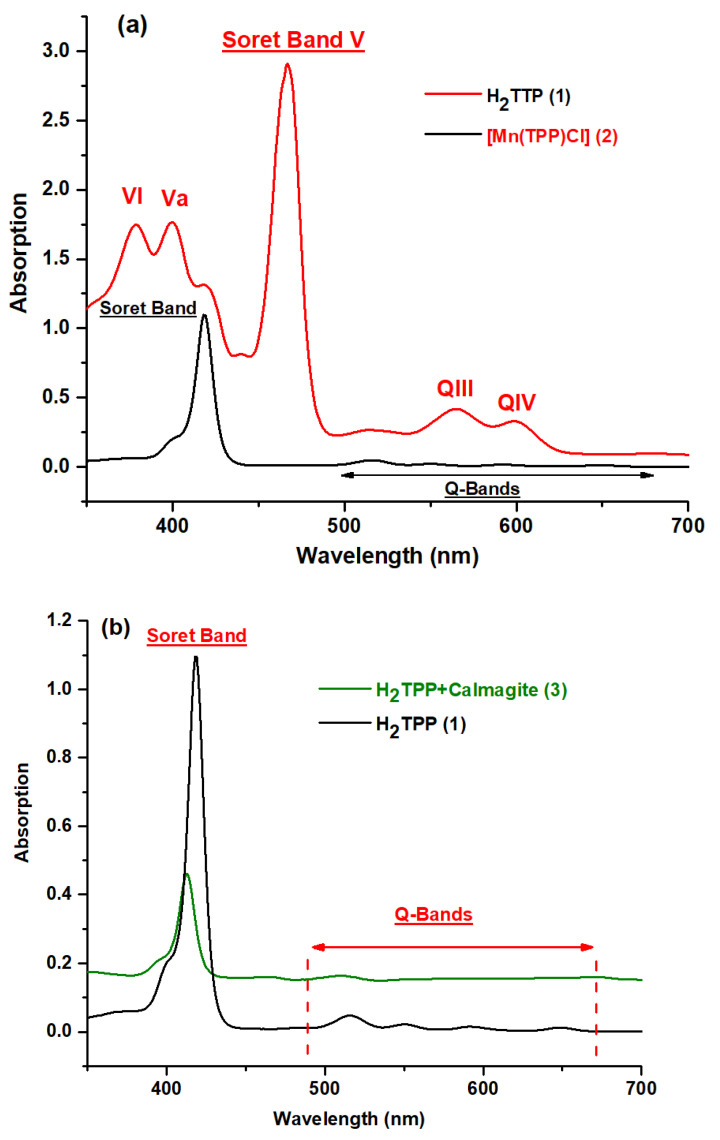

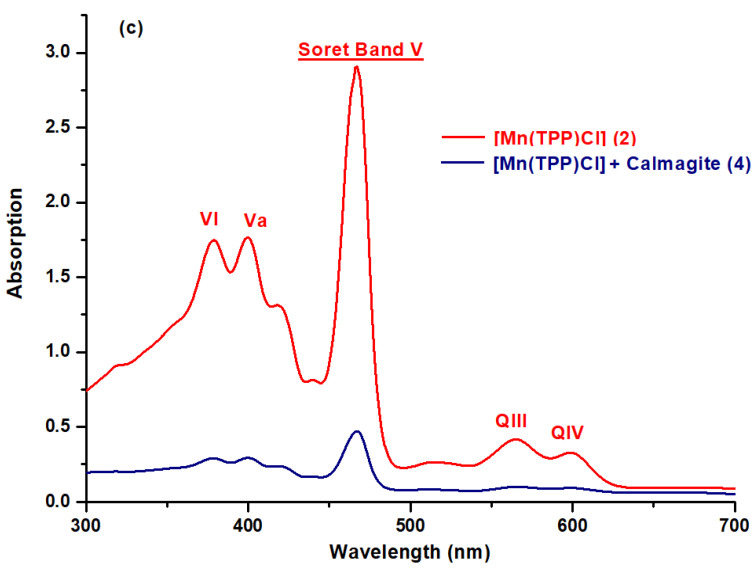
UV–vis spectra obtained in dichloromethane solutions of (**a**) H_2_TPP (**1**), [Mn(TPP)(Cl)] (**2**), (**b**) H_2_TPP + calmagite dye (**3**), and (**c**) [Mn(TPP)(Cl)] + calmagite dye (**4**).

The UV–visible spectra of H_2_TPP, H_2_TPP–calmagite, [Mn(TPP)(Cl)], and [Mn(TPP)(Cl)]-calmagite dye were recorded in dichloromethane and are shown in the scheme below. As is observed, the free base *meso*-tetraphenylporphyrin H_2_TPP depicts a Soret Band at 417 nm and Q bands at 515, 550, 595, and 646 nm (Figure 2a) [11,40,41]. The manganese(III) *meso*-porphyrins for [Mn(TPP)(Cl)] are notable for having hyper-type electronic spectra with a half-vacant metal orbital with symmetry, such as [dπ:dxz and dyz] [22,39]. The Soret or charge transfer band is the name given to the strongest band detected at 467 nm. The transfer of the porphyrin’s a1u(π) and a2u(π) orbitals to the manganese orbitals, e.g., [dπ: dxz and dyz], is responsible for this band. In addition, in the UV spectra, two other bands, which are less intense than the Soret band, are detected between 378 and 400 nm. In the visible spectra, two bands named QIII and QIV are displayed from 565 to 610 nm, while the QI and QII bands are moved into the UV–vis spectra and, consequently, they cannot be observed in the spectra (Figure 2a), along with the reduction in the number of Q-bands from four to two bands [22,42]. This confirms the insertion of the manganese into the core of free *meso*-porphyrin H_2_TPP, and this result aligns well with FT-IR data. For H_2_TPP–calmagite and [Mn(TPP)(Cl)]–calmagite, we observe a slight shifting of the position of the prominent peaks, which suggests the interaction of calmagite molecules with H_2_TPP and [Mn(TPP)(Cl)] (Figure 2b,c).

**Figure 3:**
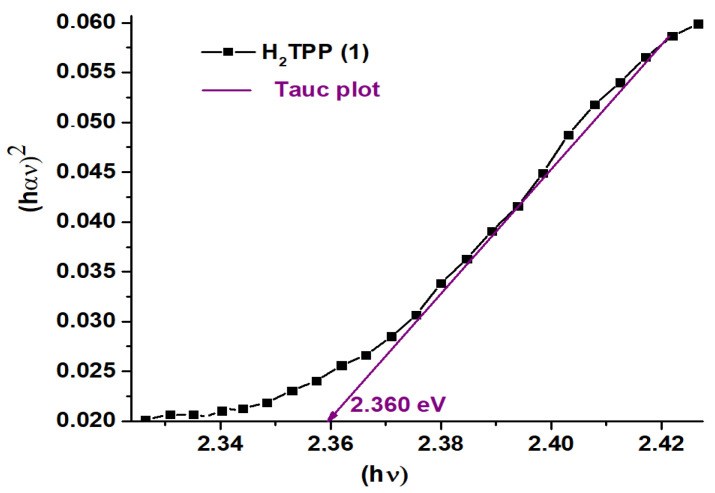

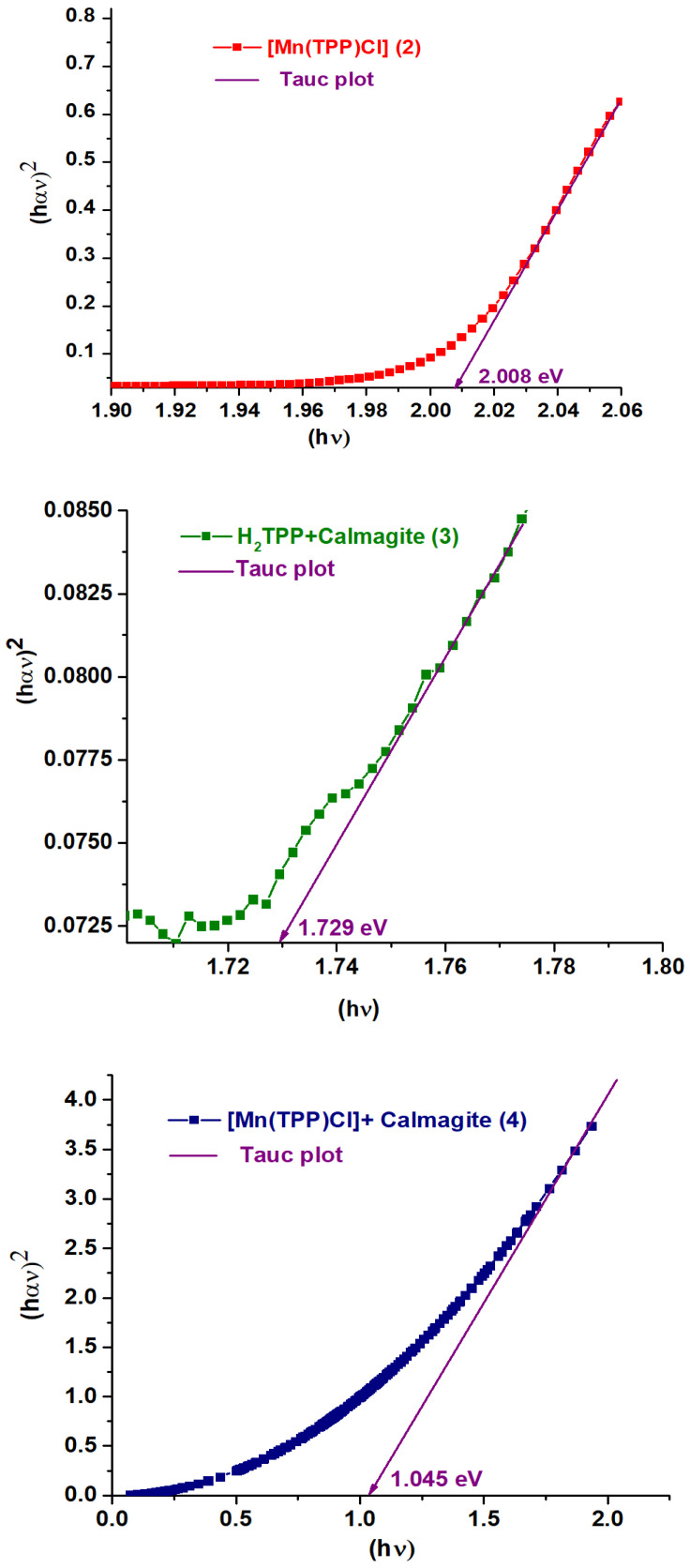
Curves of (αhυ)^2^ against the photon energy E of H_2_TPP (**1**), [Mn(TPP)(Cl)] (**2**), H_2_TPP + calmagite dye (**3**), and [Mn(TPP)(Cl)] + calmagite dye (**4**). *α* is the absorption coefficient.

Figure 3 gives the curves of (αhυ)^2^ against the photon energy E of H_2_TPP (**1**), H_2_TPP + calmagite dye (**3**), [Mn(TPP)(Cl)] (**2**), and [Mn(TPP)(Cl)] + calmagite dye (**4**). The optical gap (Eg-op) values are calculated using the Tauc plot method [43,44]. The Eg-op values for **1**–**4** are 2.360 eV, 2.008 eV, 1.729 eV, and 1.045 eV, respectively, typical for semiconductors. These values are similar to other *meso*- and *metallo*-porphyrin compounds [45].

#### 2.1.3. SEM Features

**Figure 4:**
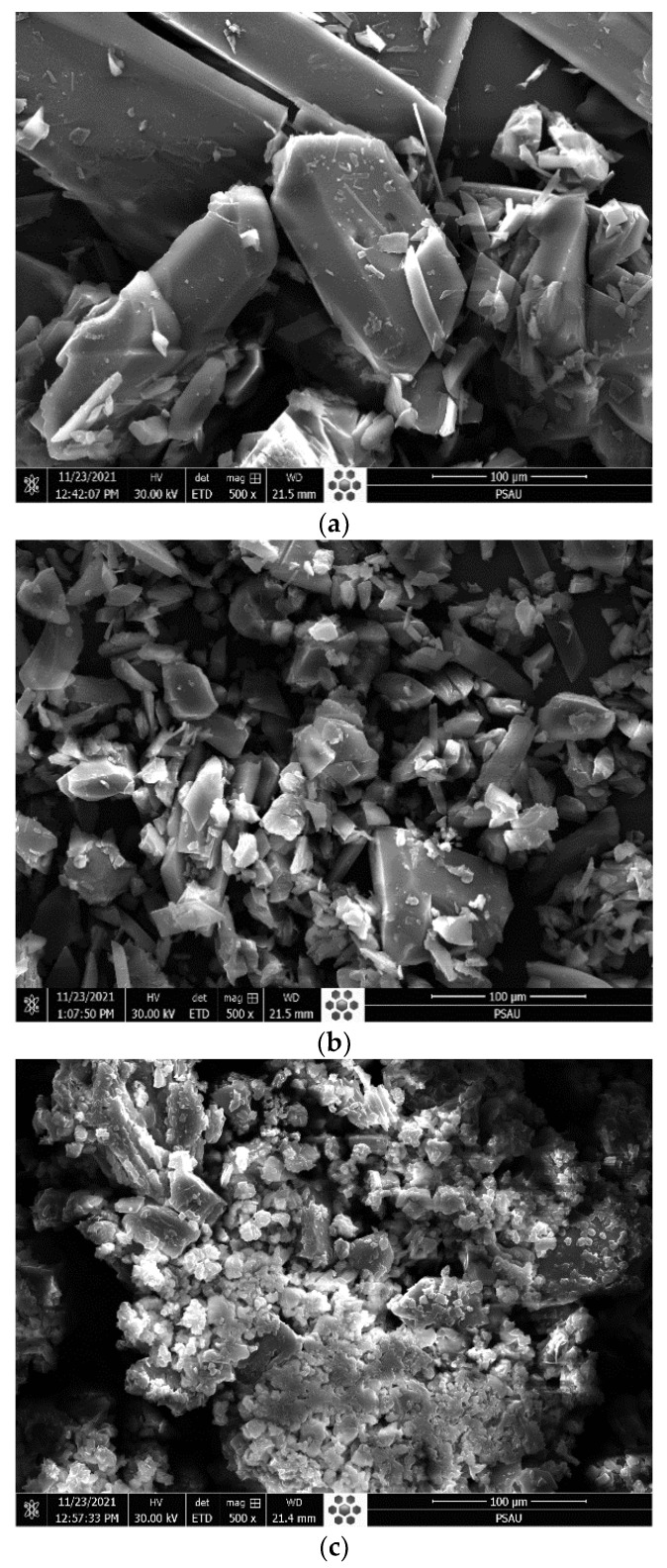

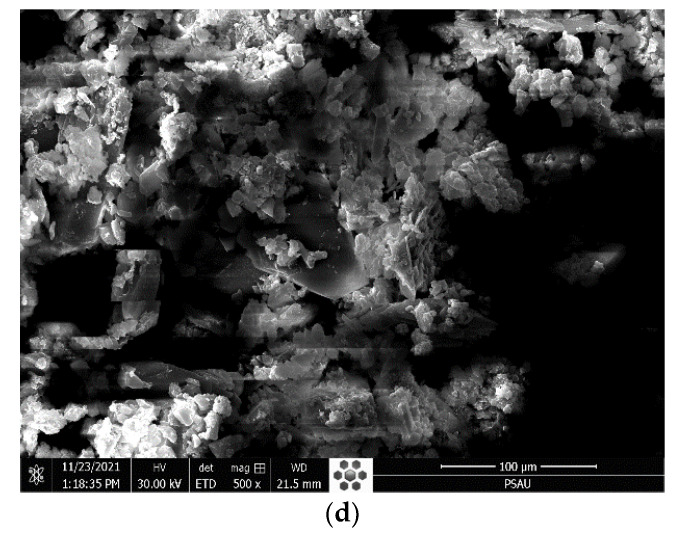
SEM pictures of (**a**) H_2_TPP, (**b**) H_2_TPP + calmagite dye, (**c**) [Mn(TPP)(Cl)], and (**d**) [Mn(TPP)(Cl)] + calmagite dye.

Figure 4 gives the SEM image of H_2_TPP and [Mn(TPP)(Cl)] compounds before and after their interaction with calmagite dye. As is observed, H_2_TPP exhibits smooth particles with irregular and different shapes. After interaction with the azoic dye molecules, these particles become too small and well swollen. The same observation was also made for the compound [Mn(TPP)(Cl)]. This observation confirms that the prepared compounds reacted strongly with calmagite molecules through the reactive groups existing in each structure.

**Scheme 1:**
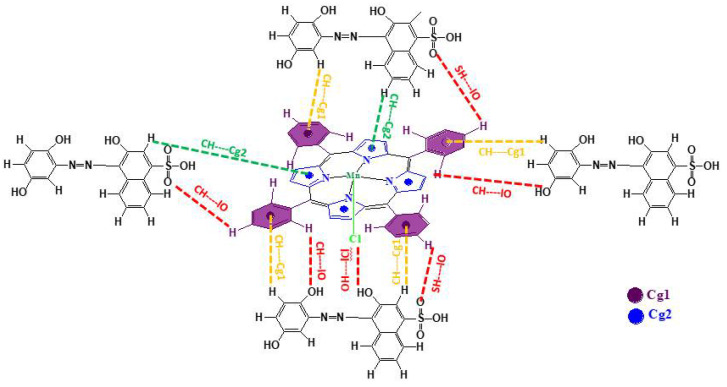
Probable interactions between calmagite dye and chloro(*meso*-tetrakis(phenyl)porphyrin) manganese (III).

Scheme 1 gives the probable interactions between calmagite dye and chloro(*meso*-tetrakis(phenyl)porphyrin) manganese(III). [Mn(TPP)Cl] and calmagite dye interacted through intermolecular O–H^…^|Cl, S–H^…^|O and C–H^…^|O hydrogen bonds and by weak C–H^…^Cg π interactions involving several centroids (Cg) of the pyrrole and *meso*-porphyrin phenyl. The proposed mechanism aligns with previously studied *meso*-tetrakis (2,4,6-trimethylphenyl) porphyrinto) zinc(II) supported by sodium alginate during the adsorption of methylene blue [46].

#### 2.1.4. XRD Patterns

**Figure 5:**
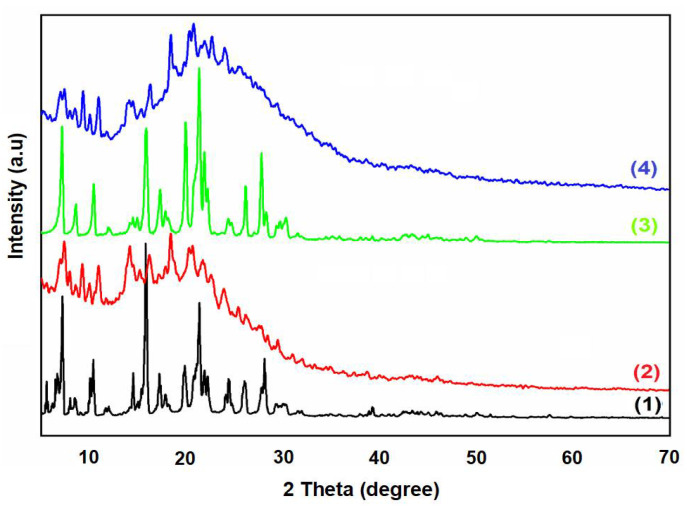
XRD patterns of H_2_TPP (**1**), [Mn(TPP)(Cl)] (**2**), H_2_TPP + calmagite dye (**3**), and [Mn(TPP)(Cl)] + calmagite dye (**4**).

Figure 5 displays the XRD patterns of H_2_TPP (**1**), H_2_TPP + calmagite dye (**3**), [Mn(TPP)(Cl)] (**2**), and [Mn(TPP)(Cl)] + calmagite dye (**4**). As is shown, the free porphyrin H_2_TPP reveals sharp peaks at 2θ that equal 8.5°, 11.50°, 15.82°, 17.95°, 20.26°, 22.95°, 25.88°, and 30.11°. [Mn(TPP)Cl] exhibits sharp peaks at 2θ equal to 7.85°, 8.90°, 11.50°, 14.60°, 16.70°, 19.50°, 20.90°, 22.20°, 24.60°, 27.50°, and 29.00°. These peaks suggest the crystalline nature of the prepared porphyrin compounds [47,48]. After the interaction of H_2_TPP and [Mn(TPP)(Cl)] with calmagite, the shifting of the position of the main peaks reveals that the dye molecules interacted with compounds H_2_TPP and [Mn(TPP)(Cl)].

#### 2.1.5. Thermal Analysis

**Figure 6:**
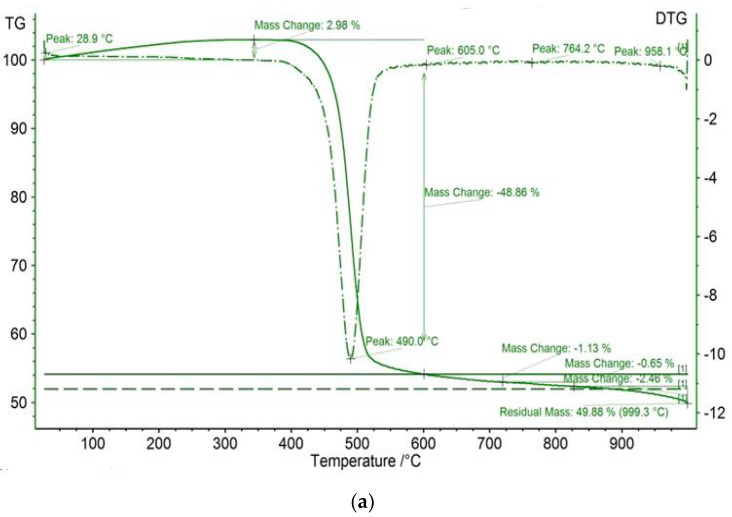

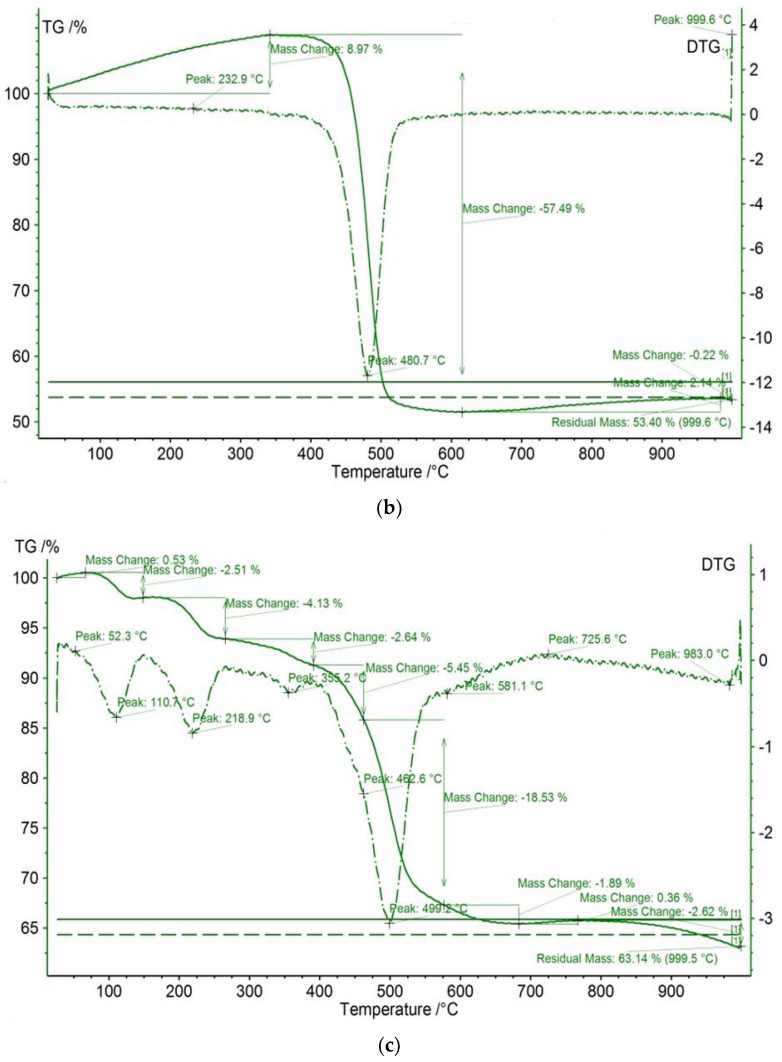

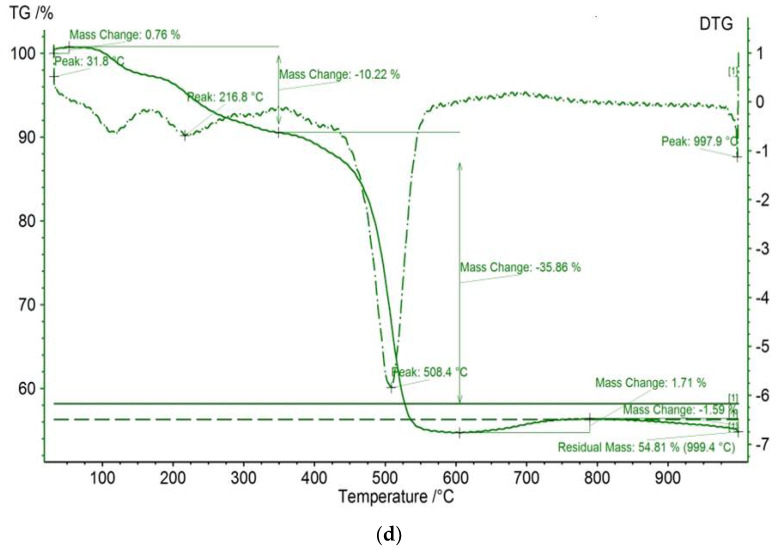
TGA/DTG curves of (**a**) H_2_TPP, (**b**) H_2_TPP + calmagite dye, (**c**) [Mn(TPP)(Cl)], and (**d**) [Mn(TPP)(Cl)] + calmagite dye.

The TGA/DTG results of *meso*-tetraphenyl porphyrin and chloro(*meso*-tetrakis(phenyl)porphyrin) manganese(III) before and after dye adsorption are shown in Figure 6. The studied compounds reveal initial thermal events, which are observed at <100°C for all investigated compounds and are attributed to adsorbed water loss [49]. The residual masses for H_2_TPP and [Mn(TPP)(Cl)] are equal to 49.88% and 63.14%, respectively. In contrast, the thermal behavior of the compound [Mn(TPP)(Cl)] appears different from that of the starting material H_2_TPP. Indeed, [Mn(TPP)(Cl)] displays a three-step degradation process. The first thermal event occurs at 110.7 °C. The second thermal event occurs at 218.9 °C. The final thermal event proceeds at 499.2 °C. The latter two events are absent for the starting material. This result confirms the incorporation of manganese into the core of free porphyrin H_2_TPP. The difference in mass remaining after the interaction between the porphyrin compounds and calmagite molecules could be explained by some new structural changes.

#### 2.1.6. XRF Characterization

X-ray fluorescence (XRF) spectroscopy data of the relative abundance of elements present in the prepared [Mn(TPP)Cl] are depicted in Table 1. As is shown, the manganese metal is present in the studied compound with a high relative amount of 70.59%. We also note the presence of other elements, such as Cl (28.60%), Co (0.706%), Nb (0.044%), and Mo (0.035%). The obtained results confirm the incorporation of manganese into H_2_TPP.

### 2.2. Degradation of Calmagite in the Presence of [Mn(TPP)(Cl)]

#### 2.2.1. Effect of Experimental Conditions

**Figure 7:**
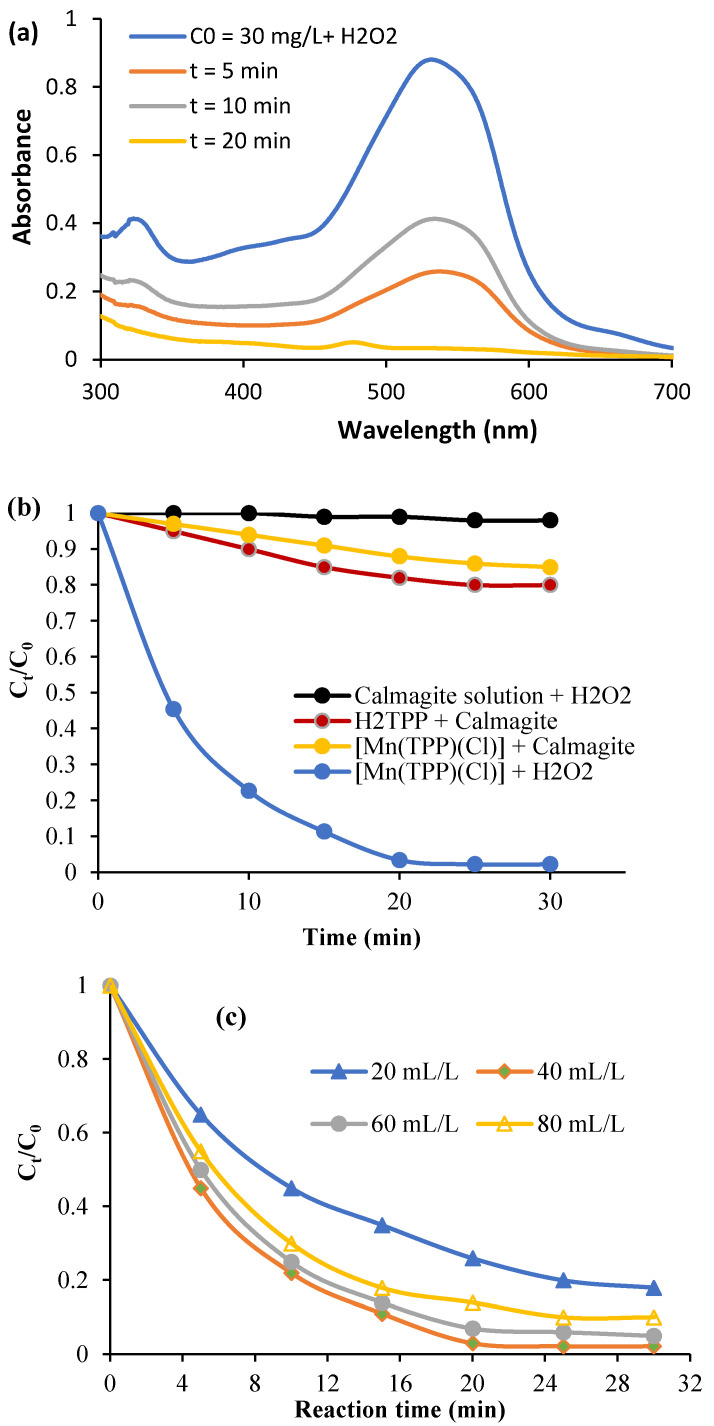

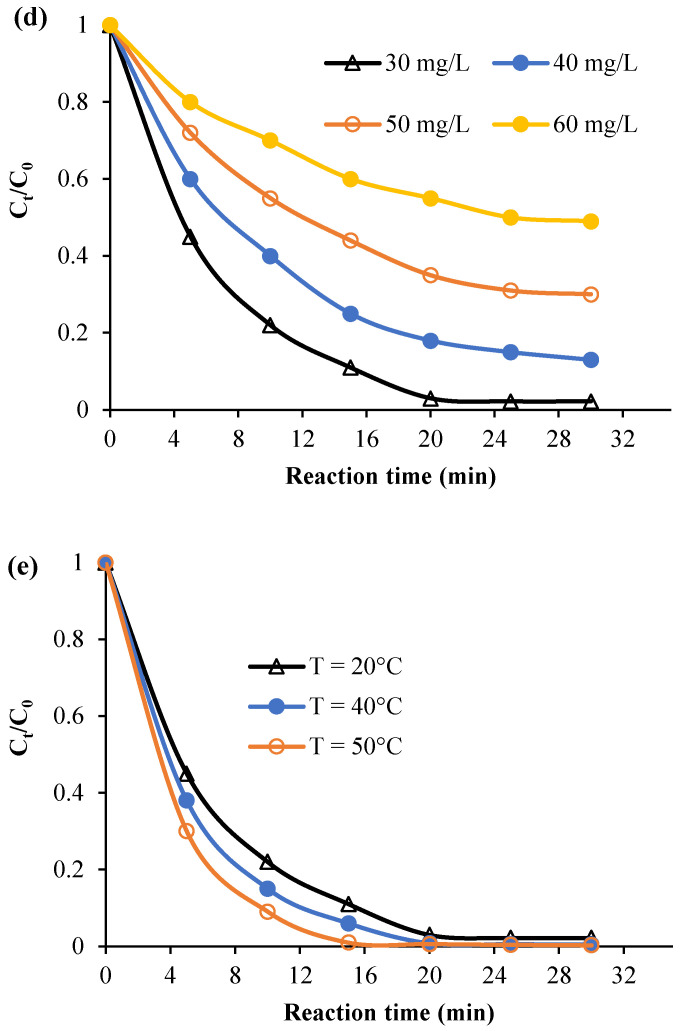
(**a**) Spectrum showing the evolution of absorbance versus time, (**b**) evolution of C_t_/C_0_ in the existence of various systems, (**c**) effect of H_2_O_2_, (**d**) starting calmagite concentration, and (**e**) temperature.

The effect of contact time on the oxidative degradation of calmagite dye solution (T = 20 °C, pH = 6, C0 = 30 mg/L, and H_2_O_2_ = 40 mL/L) is reported in Figure 7a. First, it is noted that the calmagite solution is initially not affected by the presence of H_2_O_2,_ and no degradation yield is recorded during the investigated period of time. In addition, the free porphyrin exhibits only a small attraction to calmagite, which is explained by weak bonding. In contrast, the calmagite solution is completely degraded in the presence of the system [Mn(TPP)(Cl)]/H_2_O_2_ after 25 min of reaction (Figure 7b). The azo groups of calmagite are easily degraded due to the combination of the radical OH∙ and [Mn(TPP)(Cl)].

Indeed, the mechanistic pathway of the decomposition of calmagite in the presence of the proposed system [Mn(TPP)(Cl)]/H_2_O_2_ could comprise the following steps. The first step involves the complexation of the azo calmagite dye molecules to [Mn(TPP)(Cl)], followed by peroxymetalation of the azo moiety. Then, the resulting complex could decompose to azoxy product. The degradation of the azoxy product could further afford quinolinones and diazonium salts, as established in the literature [50].

Figure 7c describes the effect of the variation in H_2_O_2_ dose on the degradation process (C0 = 30 mg/L, T = 20 °C, pH = 6). The oxidative degradation yield reaches its maximum when the dose of H_2_O_2_ is equal to 40 mL/L. These results show that using high dosages of H_2_O_2_ (more than 40 mL/L) decreases the degradation yield. Indeed, a high dose of H_2_O_2_ in calmagite solution undergoes self-quenching of OH∙, which produces HO_2_∙ radicals [51,52,53,54]. Figure 7d gives the effect of the variation in the initial calmagite concentration on the degradation yield (T = 20 °C, H_2_O_2_ = 40 mL/L, pH = 6). As is observed, the degradation yield falls with the rise in the starting calmagite concentration. At concentration = 60 mg/L, the degradation yield decreases to 51%. This result confirms that there is an optimum dose of H_2_O_2_ for each initial calmagite concentration. In addition, the degradation yield is enhanced with the rise in temperature (Figure 7e). This trend indicates that theta degradation is favored at high energy values. 

#### 2.2.2. Kinetic Modeling and Thermodynamic Investigation

Theoretical kinetic equations including zero-, first- and second-order reaction kinetics [55] were used to understand the mechanism of the degradation of calmagite using [Mn(TPP)(Cl)].

(1) $$C_{t}=C_{0}-k_{0}t$$

(2) $$C_{t}=C_{0}.e^{-k_{1}t}$$

(3) $$\frac{1}{C_{t}}=\frac{1}{C_{0}}+K_{2}t$$

where

C_t_ and C_0_ are instant and starting calmagite concentrations.

k_0_, k_1_, and k_2_ are the kinetic-rate constants of zero-, first-, and second-reaction kinetics, respectively.

**Figure 8:**
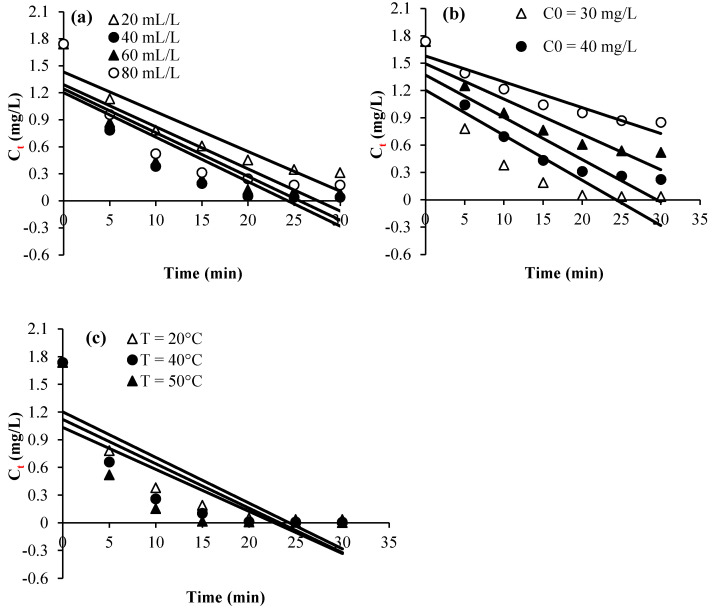
Experimental data modeling through zero-order kinetic equation: (**a**) H_2_O_2_ concentration, (**b**) dye concentration and (**c**) Temperature.

**Figure 9:**
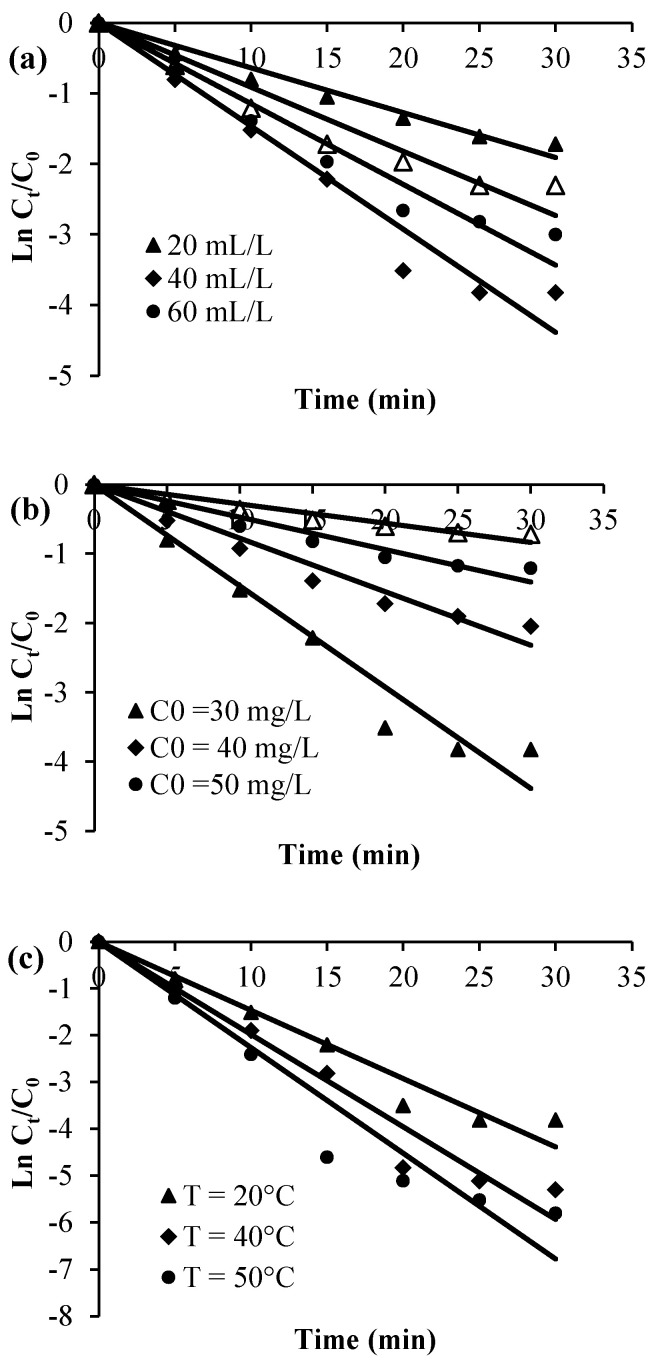
Experimental data modeling through first-order kinetic equation: (**a**) H_2_O_2_ concentration, (**b**) dye concentration and (**c**) Temperature.

**Figure 10:**
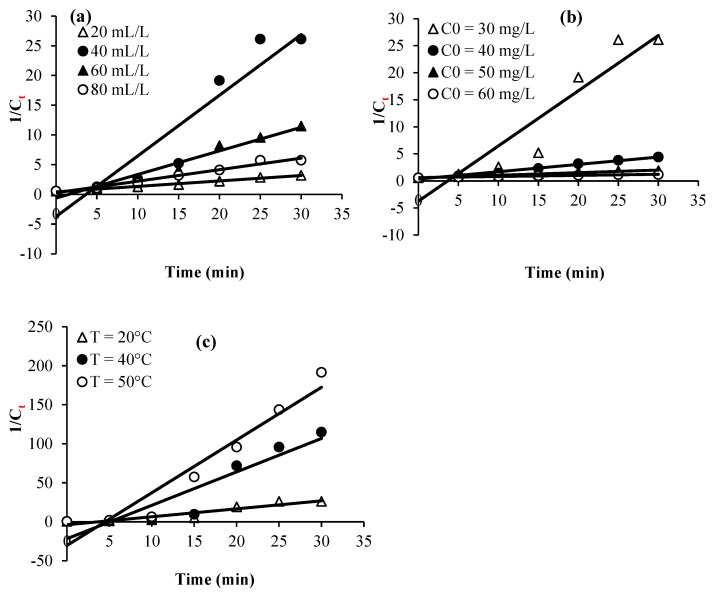
Experimental data modeling through second-order kinetic equation: (**a**) H_2_O_2_ concentration, (**b**) dye concentration and (**c**) Temperature.

Plots of C_t_, Ln (C_t_/C_0_), and 1/C_t_ against time for the various considered experimental parameters (H_2_O_2_ dose, calmagite concentration, and temperature) are given in Figure 8, Figure 9 and Figure 10. Table 2 summarizes the regression coefficients and apparent kinetic rate parameters. First-order reaction kinetics display regression coefficients (R^2^) ranging from 0.93 to 0.99, which are much higher than those obtained using second-order (0.88 < R^2^ < 0.96) and zero-order (0.59 < R^2^ < 0.90) kinetic equations. Thus, the results suggest that the degradation of the calmagite solution using [Mn(TPP)(Cl)]/H_2_O_2_ fitted well to the first-order reaction model.

The calculated kinetic rate constants obtained at the studied temperature range are listed in Table 2 and were used to determine either the thermodynamic parameters or the activation energy. The activation energy, abbreviated as (Ea, kJ mol^−1^), was determined following the Arrhenius equation [56]. 

Further, the parameters ΔS° (J mol^−1^ K^−1^) and ΔH° (kJ mol^−1^) were calculated using Equation (4) [57]:

(4) $$Ln\left(\frac{K}{T}\right)=Ln\left(\frac{K_{b}}{h}\right)+\frac{\Delta S{}^{\circ}}{R}-\frac{\Delta H{}^{\circ}}{RT}\;$$

**Figure 11:**
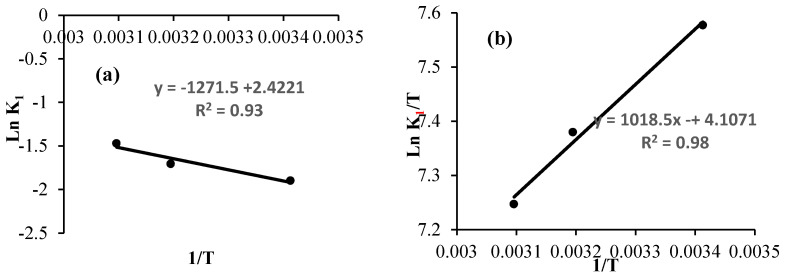
(**a**) Arrhenius plot and (**b**) plot of Ln (K_1_/T) versus the inverse of the temperature for the degradation of calmagite solution.

The activation energy was calculated from the plot representing the values of Ln K1 against 1/T (Figure 11a). However, plots of Ln (K1/T) vs 1/T were used to determine the values of ΔS° and ΔH° (Figure 11b). The free activation energy (kJ mol^−1^) was calculated using Equation 5:

(5) $${∆G}^{{}^{\circ}}={∆H}^{{}^{\circ}}-{T∆S}^{{}^{\circ}}$$

The entropy of the system is equal to −163.3 J mol^−1^ K^−1^. This negative value explains the reduction in the disorder during the process of degradation. The enthalpy is equal to 8.46 kJ mol^−1^. This positive value confirms the endothermic nature of the system.

The calculated value of Ea is equal to 10.57 kj mol^−1^, which is considered a low value. This result suggests that the decomposition of calmagite solution can be effectively achieved using the [Mn(TPP)(Cl)]/H_2_O_2_ system. Notably, the calculated Ea value agrees with results obtained during the oxidative degradation of various organic compounds utilizing some other prepared catalysts in the literature. For instance, [bis (2-methylallyl-1,5-cycloctadienne) ruthenium (II)–chitosan] was used for the oxidative degradation of calmagite and acid blue 25 solutions. The results showed that the Ea values varied from 11.16 to 27.028 kJ mol^−1^ [58]. The oxidative degradation of Eriochrome blue black b and calmagite solutions using chiral Ru(II) and Pd(II) complexes gave an Ea of 31.091 kJ mol^−1^ [51]. The Ea value calculated during the oxidative degradation of Eriochrome blue black b using palladium–oxazoline was 16.66 kJ mol^−1^ [50]. The Ea values were found to vary between 17 and 26 kJ mol^−1^ for the degradation of nitrophenol using palladium–chitosan [59]. The Ea calculated during the degradation of 4-nitrophenol using titania suspension was equal to 8 kJ mol^−1^ [60].

## 3. Materials and Methods

### 3.1. Chemicals and Reagents

Calmagite (Chemical formula: C_17_H_14_N_2_O_5_S, M. W = 385.37 g/mol, λ_max_ in water = 538 nm, purity = 60.0%) was purchased from Sigma Aldrich (St. Luis, MO, USA). Diluted solutions of H_2_SO_4_ (97%) and NaOH (reagent grade, 97%, powder) were used to regulate pH values. Aqueous colored solutions were prepared using distilled water.

### 3.2. Synthesis of Chloro(*meso*-tetrakis(phenyl)porphyrin) Manganese(III)

**Scheme 2:**
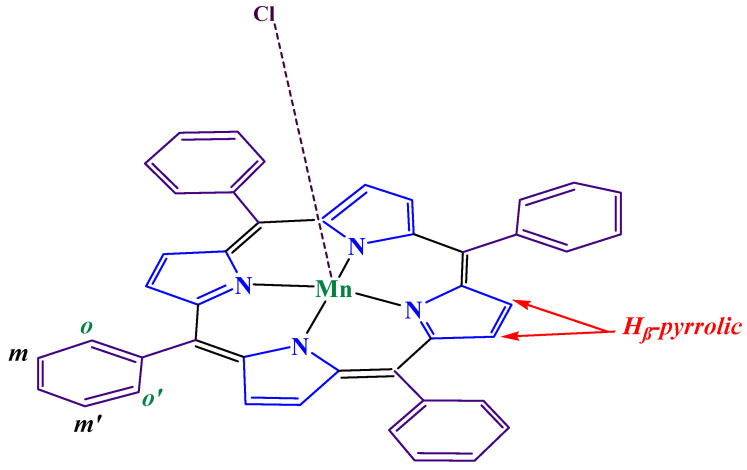
The structure of [Mn (TPP)(Cl)].

The synthesis of [Mn(TPP)Cl] (**2**) was carried out in two stages: (**i**) The Alder and Longo technique [45] was used to prepare *meso*-tetraphenylporphyrin (H_⁠2_TPP) (**1**) as the first step. (**ii**) In order to generate [Mn(TPP)Cl] (**2**), the metal must be inserted into the free porphyrin H_2_TPP in the second step. The obtained compound, shown in Scheme 2, was analyzed using IR and elemental techniques. Anal. Calc. For [Mn(TPP)(Cl)]: C_44_H_28_N_4_Mn (667.66 g/mol). C, 47.85; H, 2.31; N, 5.01%. Found: C, 46.91; H, 2.45; N, 4.95%. IR (cm^−1^): 3140–2095; ν(CH) porphyrin, 1600–1400; ν(C=N) porphyrin, 1610; ν(C=C) porphyrin, 1217–1170; δ(C-C) porphyrin, 1005; δ(CCH) porphyrin, 811–696; ν(C-C) phenyl group. UV–visible (CH_2_Cl_2_): λ_max_ nm (logε): 378(5,44), 400(5,30), 467(5.70), 565 (4.89), and 610 (4.50).

### 3.3. Sample Characterization

The Perkin Elmer Spectrum Two ATR-FTIR with a UATR unit was utilized to identify the chemical structures of the prepared compounds and evaluate any changes that resulted from their interaction with dyes. FT-IR spectra were studied in 32 scans from 400 to 4000 cm^−1^ at a resolution of 4 cm^−1^. UV–vis measurement and titration were acquired using WinASPECT PLUS (https://winaspect-plus.software.informer.com/4.0/#google_vignette, accessed on 11 October 2024). A JEOL JSM-5400 Scanning Electron Microscope (JEOL, Peabody, MA, USA) was used to examine the morphological features of the studied samples. The samples were coated with gold using a vacuum sputter coater to improve the quality of the pictures and increase the conductivity of the samples. The acceleration voltage was 20 kV. A PANalytical X’Pert PRO MPD device (Malvern Panalytical Ltd., Westborough, MA, USA) was used to evaluate the XRD patterns, which were examined in the 2-theta range of 10 to 90. TGA/DTA analysis (NETZSCH STA 449F3 instrument, NETZSCH, Burlington, MA, USA) was performed at a heating rate of 10°/min, in air flow, using platinum crucibles. An energy dispersive X-ray fluorescence spectrometer (EDXRF) unit, JSX-3202-M, was used to analyze the elements present in porphyrinic complexes.

### 3.4. Oxidative Degradation of Dyes

Erlenmeyer flasks with 0.001 g of [Mn(TPP)(Cl)] and 10 mL of calmagite were used for the degradation studies. A measured dose of H_2_O_2_ was then added. At 125 rpm, the solution was continuously swirled. At the completion of each experiment, the contents of each flask were filtered, and a UV–Vis spectrophotometer was used to measure each solution’s absorbance at 538 nm. Experiments were evaluated at various temperatures (20 to 50 °C), dye concentrations (30 mg/L to 60 mg/L), contact periods (0 to 60 min), and H_2_O_2_ dosages (20–80 mL/L). 

## 4. Conclusions

To summarize, H_2_TPP and [Mn(TPP)(Cl)] were successfully synthesized and thoroughly characterized. The compounds were analyzed using several analytical techniques, including FT-IR, SEM, UV, TGA/DTA, and XRD. Manganese(III) *meso*-porphyrins revealed a hyper-type electronic spectra with a half-vacant metal orbital with symmetry. The thermal behavior of [Mn(TPP)(Cl)] changed significantly compared to the starting H_2_TPP. The XRD result exhibited the crystalline nature of the prepared porphyrin compounds. The SEM showed smooth particles with irregular and different shapes. The XRF results confirmed the incorporation of the manganese into the core of free porphyrin H_2_TPP. The prepared compounds were used as catalysts for the oxidative degradation of calmagite using H_2_O_2_. The degradation mechanism depended on reaction time, H_2_O_2_ dose, initial dye concentration, and temperature. The calmagite solution was decolourized entirely in the presence of the system [Mn(TPP)(Cl)]/H_2_O_2_ with the following conditions: pH = 6, temperature = 20 °C, C_0_ = 30 mg/L, and H_2_O_2_ = 40 mL/L. The calculated low activation energy Ea (10.55 Kj/mol) demonstrated the efficiency of the proposed system. Given the synthesis method and exceptional catalytic characteristics, the produced catalyst has the potential to be used in water purification. Because the preparation of these catalysts was simple and due to their ease of recovery, future experiments might be expanded to design new catalytic systems and identify the by-products obtained during the catalytic decomposition.

## Figures and Tables

**Table 1:** Relative abundance of elements present in [Mn(TPP)Cl].

	m/m (%)
Elements	[Mn(TPP)Cl]
Mn	70.59
Cl	28.60
Co	0.706
Nb	0.044
Mo	0.035
In	0.016

**Table 2:** A summary of the calculated kinetic rates and thermodynamic parameters for the degradation of calmagite using [Mn(TPP)(Cl)]/H_2_O_2_.

Kinetics	Zero-Order		First-Order		Second-Order	
	K_0_	R^2^	K_1_	R^2^	K_2_	R^2^
H_2_O_2_ amount (mL)						
20	1.43	0.86	0.06	0.99	0.44	0.96
40	1.2	0.73	0.14	0.95	3.72	0.88
60	1.24	0.75	0.11	0.96	0.60	0.93
80	1.30	0.76	0.09	0.97	0.28	0.93
Calmagite concentration (mg/mL)						
30	1.2	0.73	0.14	0.95	3.72	0.88
40	1.4	0.82	0.07	0.98	0.58	0.94
50	1.49	0.87	0.05	0.96	0.34	0.93
60	1.58	0.90	0.03	0.98	0.61	0.92
Temperature (°C)						
20	1.20	0.73	0.15	0.95	3.72	0.88
40	1.12	0.67	0.18	0.96	21.66	0.90
50	1.04	0.59	0.23	0.93	30.29	0.93
Thermodynamic parameters						
Temperature (°C)	Ea (Kj/mol)		ΔS° (j/mol/K)	ΔH° (kj/mol)		ΔG° (kj/mol)
20						56.33
40	10.57		−163.3	8.46		59.60
50						61.24

## Data Availability

Data are contained within the article.
